# Hydrophobic, Sustainable, High-Barrier Regenerated Cellulose Film via a Simple One-Step Silylation Reaction

**DOI:** 10.3390/polym15081901

**Published:** 2023-04-15

**Authors:** Goomin Kwon, Jisoo Park, Kangyun Lee, Youngsang Ko, Youngho Jeon, Suji Lee, Jeonghun Kim, Jungmok You

**Affiliations:** 1 Department of Plant & Environmental New Resources and Graduate School of Green-Bio Science, Kyung Hee University, 1732 Deogyeong-daero, Giheung-gu, Yongin-si 17104, Gyeonggi-do, Republic of Korea; 2 Department of Chemical and Biomolecular Engineering, Yonsei University, 50 Yonsei-ro, Seodaemun-gu, Seoul 03722, Republic of Korea; oio_jisoo@yonsei.ac.kr (J.P.); jhkim03@yonsei.ac.kr (J.K.)

**Keywords:** regenerated cellulose, biopolymer film, surface modification, chemical vapor deposition, organic chlorosilane, barrier properties

## Abstract

With the increasing importance of environmental protection, high-performance biopolymer films have received considerable attention as effective alternatives to petroleum-based polymer films. In this study, we developed hydrophobic regenerated cellulose (RC) films with good barrier properties through a simple gas–solid reaction via the chemical vapor deposition of alkyltrichlorosilane. RC films were employed to construct a biodegradable, free-standing substrate matrix, and methyltrichlorosilane (MTS) was used as a hydrophobic coating material to control the wettability and improve the barrier properties of the final films. MTS readily coupled with hydroxyl groups on the RC surface through a condensation reaction. We demonstrated that the MTS-modified RC (MTS/RC) films were optically transparent, mechanically strong, and hydrophobic. In particular, the obtained MTS/RC films exhibited a low oxygen transmission rate of 3 cm^3^/m^2^ per day and a low water vapor transmission rate of 41 g/m^2^ per day, which are superior to those of other hydrophobic biopolymer films.

## 1. Introduction

Petroleum-derived synthetic polymers, such as polystyrene (PS), polyethylene (PE), and polyethylene terephthalate (PET), have been the main sources of various polymer films because they are easily processable, low-cost, durable, and highly scalable materials [1,2,3]. Polymer films have numerous industrial applications, including in the food packaging industry. However, these plastic films, which exhibit poor degradability and are difficult to recycle, have given rise to significant disposal and pollution issues, which threaten human health and the environment [4,5,6]. In particular, the accumulation of traditional food packaging films made from nondegradable materials is proving to be a serious issue for the environment. To solve this problem, many efforts have been dedicated to developing natural biopolymer films that are biodegradable, sustainable, and possess good mechanical and barrier properties [7,8,9]. Despite the fact that biopolymer films provide an alternative solution to traditional food packaging films and have attracted considerable attention, they are characterized by several intrinsic disadvantages, including their low mechanical strength and poor barrier properties, which limit their use in commercial applications [10,11,12]. In particular, excellent gas and water vapor barrier properties are highly desirable for food packaging applications to ensure food safety.

Among the different nature-derived polymers, cellulose is the most abundant renewable natural polymer on Earth and is a promising biopolymer for various applications owing to its biodegradability, low cost, mechanical strength, and chemical stability [13,14,15]. Over the past decades, nanocellulose and RC materials have been extensively utilized as biodegradable, transparent, and freestanding biopolymer films [16,17,18]. Specifically, RC films are expected to replace synthetic-plastic-based food packaging films because they present the advantages of both synthetic and natural polymer films, namely high uniformity, good solution processability, excellent mechanical properties, biodegradability, and sustainability. The strong hydrogen bonds with a hydroxyl group (–OH) in cellulose are the predominant contributors to its outstanding mechanical strength and chemical stability [19,20]. On the other hand, cellulose films are hydrophilic due to the presence of a large number of hydroxyl groups on their surface, which makes them sensitive to moisture, resulting in poor oxygen and water vapor barrier properties.

A number of laboratories have been developing various strategies to fabricate hydrophobic cellulose films using layer-by-layer assembly methods, hydrophobic components, and surface modification approaches [21,22,23]. However, multilayer cellulose films obtained through layer-by-layer assembly contain petroleum-based plastic or aluminum as barrier materials, which limits their recyclability because of the difficulty in separating cellulose from the plastic or metallic layer [24,25]. Traditional barrier materials, such as silica, alumina, or metals, are detrimental to the recyclability of biopolymer films. Furthermore, the introduction of hydrophobic components results in aggregation and agglomeration in the cellulose matrix due to the poor compatibility between hydrophobic additives and hydrophilic cellulose [26,27]. Agglomerate structures lead to the lower mechanical strength and the nontransparency of cellulose films. Various surface modification techniques based on chemical agents and fluorinated coating materials have been developed to control the wetting properties of cellulose films because of their low surface energy [28,29]. Recently, Makarov et al. reported a facile method to modify the surface layer of cellulose paper with a N-methylmorpholine-N-oxide (NMMO) solvent [30]. Deep interactions between NMMO and cellulose fiber can lead to the strong swelling and dissolution of cellulose, leading to a decrease in air permeability and water absorption. Among the chemical agents, fluorinated chemicals are characterized by several major disadvantages, including their high manufacturing cost, processing problems, and their threat posed to human health and the environment [31]. Therefore, in order to overcome the disadvantages of fluorinated chemicals, there are several studies that have employed organic-silicon-based compounds to adjust hydrophobicity, lower humidity sensitivity, and increase interfacial adhesion [32,33,34,35]. The use of a variety of silane coupling agents allows for easy control over surface properties. Sfax et al. investigated the interaction of silane with a cellulose fiber surface, according to various alkoxysilanes [36]. The alkoxysilanes created a monolayer of silane covalently bonded to a cellulosic substrate through a condensation reaction. Castellano et al. studied the reaction conditions between cellulose fibers and trialkoxysilane, including cyanoethyltrimethoxysilane (CES) and γ-methacryloxypropyltrimethoxysilane (MPS) [37]. Under high temperature, siloxane could form silanol groups through partial hydrolysis, which could react with OH groups of cellulose. Rutter et al. constructed hydrophobic cellulose filter paper by using a mixture of vinyltrimethoxysilane and siloxane in its hydrolyzed form [38]. Polysiloxane covalently bonded with the surface of the filter paper, which showed good stability even at high temperatures. Ganicz et al. reported a new method of paper hydrophobization with a coating agent containing a water emulsion of triethoxymethylsilane [39]. As a result of the self-condensation of alkoxysilane, silica microparticles were deposited on the surface of cellulose paper to produce hydrophobic properties, which finally led to a decrease in air permeance. Previously, we developed cellulose nanocrystal (CNC)-coated TEMPO-oxidized cellulose nanofiber (TEMPO-CNF) films for enhancing barrier properties [7]. In this study, CNCs were used as a coating material to improve the water vapor barrier properties of TEMPO-CNF film. However, we encountered high energy consumption and extra time for CNC production through acid hydrolysis.

In this study, we report a very simple, rapid, and efficient approach for developing hydrophobic RC films with good optical transparency, high mechanical strength, and good barrier properties via a one-step chemical vapor deposition (CVD) method. We developed methyltrichlorosilane (MTS)-modified RC (MTS/RC) films through a highly efficient and low-cost gas–solid reaction process with MTS, addressing the specific challenges of oxygen/water vapor permeability. The reaction of vaporous MTS with an RC surface exhibiting numerous hydroxyl groups resulted in the formation of hydrophobic RC films. Our experiments reveal that hydrophobic MTS/RC films exhibit considerably better barrier properties than pristine RC films without surface modification. To the best of our knowledge, no previous works have reported organic-chlorosilane-modified RC films with good barrier properties. Overall, the simple approach described here can enhance our ability to create hydrophobic biopolymer films with excellent barrier properties.

## 2. Materials and Methods

### 2.1. Chemicals and Materials

Cellulose powder (cotton linters, medium fiber) and MTS (purity > 99%) were purchased from Sigma-Aldrich Co. (Seoul, Korea) Lithium hydroxide (LiOH, purity = 99%), urea (purity = 98%), and ethyl alcohol (purity = 95%) were purchased from Duksan Pure Chemicals Company. DIW was purified using an EXL3 pure and ultrapure water system (VIVAGEN CO., LTD., Seongnam, Korea). All the chemicals were used without further purification.

### 2.2. Characterization and Measurements

Field-emission scanning electron microscopy (FE-SEM, AURIGA, Zeiss, Oberkochen, Germany) was used to investigate the surface morphology of the pristine RC film as well as that of the RC/MTS films. FE-SEM (JEOL, JSM-7610F plus, Tokyo, Japan) equipped with EDS was used to perform the elemental mapping and energy-dispersive spectroscopy (EDS) characterization. The mechanical strength of the films was evaluated using a texture analyzer (CT3 25 K, Brookfield, WI, USA) with a 20 g load cell at a speed of 0.5 mm s^−1^. Before measuring mechanical strength, the samples were dried in an oven at 60 °C to perfectly remove residual moisture. The size of the samples was 30 mm in length and 10 mm in width. Seven samples of each film were tested in order to determine the tensile strength and Young’s modulus. All the mechanical strength measurement were performed at room temperature (23 °C) and 10% relative humidity (RH). An ultraviolet–visible (UV–vis) spectrophotometer (Neo-S2117, Neogen, Chicor, Korea) was employed to determine the transmittance of the films. The Fourier transform infrared (FT-IR) spectra of the pristine RC film, as well as the RC/MTS-10, RC/MTS-30, and RC/MTS-60 films, were acquired using an FT-IR spectrometer (Bruker Corporation, ALPHA II, Ettlingen, Germany) in ATR mode. X-ray photoelectron spectroscopy (XPS) was conducted using a K-alpha (Thermo scientific Inc., Oxford, UK) to investigate the chemical composition. Photoelectrons were generated by a monochromatic Al X-ray source (Al Kα line = 1486.6 eV) with a spot diameter of 400 µm at room temperature and a pressure of 4.8 × 10^−7^ Pa. The survey spectra were collected over the energy range of 0–900 eV. Origin Pro (version 8.5) software was utilized to analyze raw data. The binding energy was calibrated using the C1s at the 284.8 eV peak.

### 2.3. Fabrication of the RC Films

To prepare an 8 wt% cellulose solution, 4 g of cellulose was dissolved in 50 g of an aqueous solution composed of 4.6 wt% LiOH, 15 wt% urea, and 80.4 wt% deionized water (DIW) while stirring for 1 h. The prepared cellulose solution was immediately and rapidly frozen using liquid nitrogen. After the complete dissolution of the cellulose, it was thawed and centrifuged at 4000 rpm for 3 min to remove air bubbles from the dissolved cellulose solution. The prepared cellulose solution was poured into a glass mold (9 cm × 9 cm with a thickness of 1 mm) and immersed in an ethanol bath for 2 h to obtain an RC hydrogel. After 2 h, the obtained RC hydrogel was washed with excessive DIW for 12 h to remove the residual reagent. The RC hydrogel was dried at 40 °C and 90% RH in thermo-hygrostat to obtain the RC films.

### 2.4. Preparation of the Hydrophobic MTS-Modified RC Films

To fabricate the hydrophobic RC films, a vial containing the MTS solution (1 mL) and the dried RC film (7 cm × 7 cm with a thickness of 70 µm) was placed in a desiccator. The desiccator was then placed in an oven at 60 °C to vaporize the MTS. The gas–solid reaction was performed for 10, 30, or 60 min. Here, the MTS-modified RC films are referred to as MTS/RC-10, MTS/RC-30, and MTS/RC-60, respectively. Finally, the RC/MTS films were subsequently dried in a fume hood for 2 h to remove the unreacted silane as well as the by-product, hydrochloric acid, before use [40,41,42].

### 2.5. Contact Angle Measurements

The contact angles of pristine RC and MTS/RC films were measured using a contact angle analyzer (Phoenix 300, SEO, Suwon, Republic of Korea) at room temperature (23 °C) using the static method. A 10 µL DIW drop was placed on the surface of the pristine RC and MTS/RC films. Each sample was fixed on a microslide glass with 3M tape to prevent movement of the flat surface during wetting. Immediately afterward, the contact angle image was acquired using a charge-coupled device (CCD) camera. The contact angle measurement for each film was repeated at least three times.

### 2.6. Oxygen Transmission Rate (OTR) and Water Vapor Transmission Rate (WVTR)

To evaluate the oxygen barrier properties, the oxygen transmission rate (OTR) was measured using an oxygen permeation analyzer (OX-TRAN Model 2/21, Mocon, Brooklyn Park, MN, USA) in the range of 0.05–2000 ccm^−3^ day^−1^, according to the ASTM D 3895 standard. The pristine RC and MTS-modified RC films (sample area: 1 cm^2^) were mounted on an aluminum mask, and the OTR measurements were carried out under conditions of 23 °C and 0% RH. One side of the film was exposed to nitrogen gas, and the opposite side was exposed to flowing nitrogen gas (10 sccm). The amount of permeated oxygen was measured for 30 min using a detector when the transmission reached a steady state. Fifteen cycles were repeated, and it took 10 h to complete one test. The WVTR was measured following the ASTM E96 standard. Before measuring WVTR, calcium chloride (CaCl_2_) was baked on a hot plate at 120 °C for 6 h to perfectly remove residual the water existing in CaCl_2_. Additionally, baked CaCl_2_ was poured into a snap ring vial with a diameter of 12 mm, and the cap of the vial was sealed with filter paper, the pristine RC film, and the MTS/RC films. The sealed vials were stored at 23 °C and 50% RH for 24 h, and the weight of CaCl_2_ was measured to calculate the WVTR. Five samples of each film were tested in order to determine the WVTR.

## 3. Results

**Figure 1:**
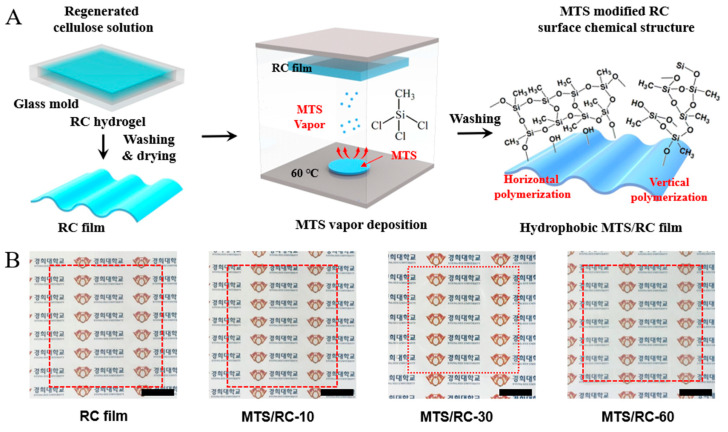
(**A**) Fabrication of the hydrophobic MTS/RC films via CVD. Firstly, the cellulose solution was poured into a glass mold and coagulated into RC films. Next, the dried RC film was placed in a desiccator, and MTS was vapor-deposited on the surface of the RC film for 10, 30, or 60 min. (**B**) Photographs of the pristine RC film and the MTS/RC-10, MTS/RC-30, and MTS/RC-60 films. Inset scale bar: 2 cm. Thickness of the RC and MTS/RC films: 70 µm.

In this study, the MTS/RC films were fabricated using RC as a biopolymer film substrate and MTS as a hydrophobic coating layer. Figure 1A provides a schematic of the fabrication process for the hydrophobic MTS-modified RC films via the gas–solid silylation reaction. Firstly, the cellulose solution was poured into a glass mold and soaked in an ethanol coagulation bath to form the RC hydrogels. The size of RC films can be scaled by controlling the size of the glass mold [43,44,45]. After complete drying, the RC hydrogels were transformed into RC films with a thickness of 70 µm. Next, the RC film and MTS were placed in a closed chamber. Upon heating at 60 °C, the MTS reacted with the hydroxyl groups of the RC surface to form covalently attached hydrophobic layers [46,47]. Importantly, alkyltrichlorosilanes provide a relatively fast covalent coupling reaction without prehydrolysis steps, and they can thus readily couple with the hydroxyl groups of an RC surface through a condensation reaction [48,49]. To investigate the optical, mechanical, and wetting properties of the obtained MTS/RC films as a function of the MTS deposition time, MTS/RC films with different deposition times (namely 10, 30, or 60 min) were fabricated (MTS/RC-10, MTS/RC-30, and MTS/RC-60, respectively). As shown in Figure 1B, the pristine RC film and the MTS/RC films were found to be highly optically transparent. Interestingly, MTS/RC-60, which showed no evident thickness change compared with the pristine RC film, still exhibited optical transparency.

**Figure 2:**
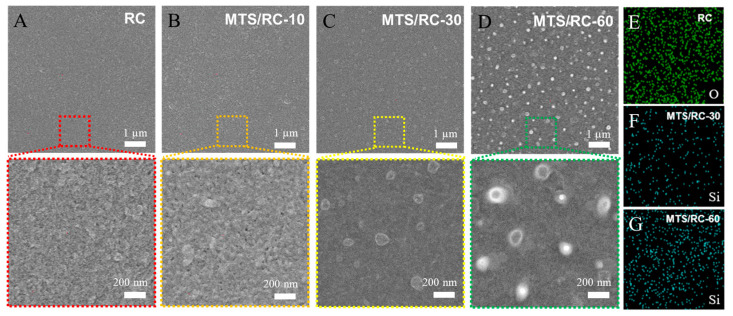
Top-view FE-SEM images of (**A**) the pristine RC film and (**B**–**D**) the MTS/RC-10, -30, and -60 films as a function of the MTS deposition time (10, 30, and 60 min). EDS elemental mapping images of (**E**) the pristine RC film, (**F**) MTS/RC-30, and (**G**) MTS/RC-60.

Figure 2 shows top-view FE-SEM images of the pristine RC film and the MTS/RC-10, MTS/RC-30, and MTS/RC-60 films. The pristine RC film shows a smooth morphology without any aggregated particles (Figure 2A). Importantly, the surface morphology of the MTS/RC-10 film is very similar to that of the pristine RC film, suggesting that MTS did not fully cover the RC surface (Figure 2B). However, as the MTS deposition time increased to 30 and 60 min, a different surface morphology with aggregated nanoparticles was clearly observed, probably due to the hydrolytic polycondensation of MTS (Figure 2C,D) [50,51]. As shown in Figure 2A, MTS could mainly react with the hydroxyl groups present on the surface of the RC film, because the RC film did not have a porous structure. With increasing reaction time, the polymeric siloxane structure was created through horizontal and vertical condensation to form an aggregate morphology on the top of the RC surface (Figure 1A) [52,53]. These results indicate that the MTS layers fully covered the RC surface when the MTS deposition time was 30 or 60 min. EDS analysis was performed to confirm the elemental composition of the RC and MTS/RC films. The element O was clearly detected on the pristine RC film due to its abundance in cellulose (Figure 2E). Notably, the presence of Si was only found on the MTS/RC-30 and -60 films (Figure 2F,G), indicating the successful grafting of silane groups onto the MTS/RC films.

**Figure 3:**
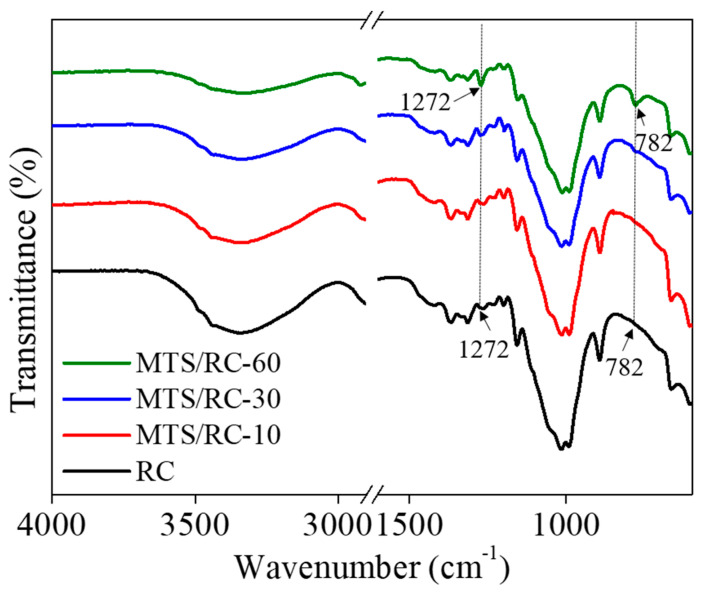
FT-IR spectra of the pristine RC film and the MTS/RC films with different MTS deposition times (10, 30, and 60 min).

To further confirm the introduction of MTS into the RC films, we carried out FT-IR analyses on the pristine RC film and the MTS/RC films obtained after different deposition times (Figure 3). The broad peak of the –OH stretching vibration at around 3300 cm^−1^ gradually disappeared with an increasing MTS deposition time, indicating that the surface hydroxyl group of cellulose reacted with MTS [31,42,54]. Compared with the pristine RC film, the MTS/RC films exhibited new peaks at 1272 and 782 cm^−1^, which could be assigned to the asymmetric stretching vibrations of Si–CH_3_ and the characteristic vibrations of Si–O–Si, respectively [46,55,56,57]. In contrast to MTS/RC-30 and -60, two peaks at 1272 and 782 cm^−1^ were not clearly detectable for the MTS/RC-10 film, probably due to the lack of silanization. Importantly, with an increase in the deposition time of MTS, the MTS/RC films showed more distinct peaks at 1272 and 782 cm^−1^. These results indicate that a stronger silicone coating was formed on the surface of the MTS/RC-60 films. These FT-IR data are in line with the FE-SEM images reported in Figure 2.

**Figure 4:**
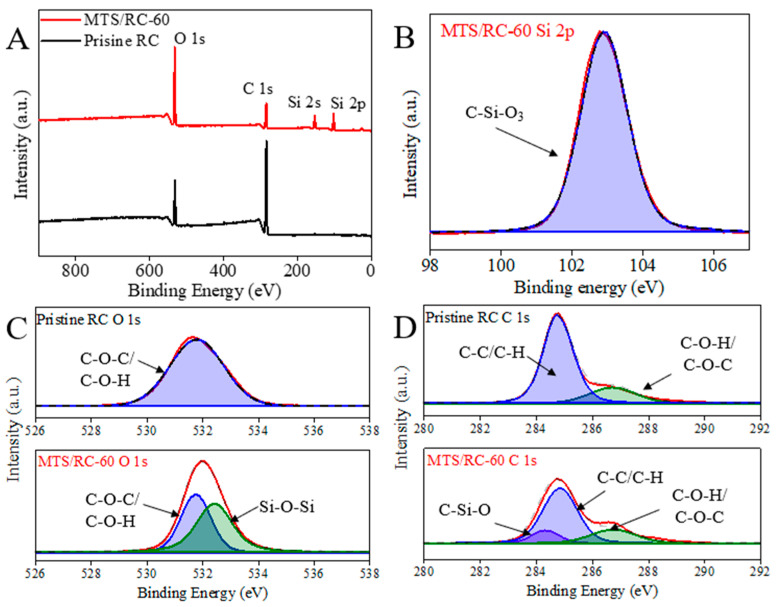
(**A**) XPS spectra of pristine RC and MTS/RC-60 film. (**B**) High-resolution spectrum Si 2p of MTS/RC-60 film. (**C**,**D**) High-resolution spectra O 1s and C 1s of pristine RC and MTS/RC-60 films.

The surface element compositions of pristine RC and MTS/RC films were further examined with XPS analysis (Figure 4A–D). For the pristine RC, only the peaks of O and C, which are present in cellulose, were found without any impurities (Figure 4A). In contrast to the pristine RC film, two new peaks at 101.6 and 151.0 eV were observed in the spectrum of MTS/RC-60, which we attributed to Si 2p and Si 2s peaks. In addition, a clear peak in the high-resolution spectra XPS of Si 2p at 102.8 eV could be attributed to C–Si–O_3_ (Figure 4B) [42,58]. MTS that contains hydrolysable groups such as chloride can react with water to produce intermediate silanols, which can further react with other silanols or hydroxyl groups present on the surface of RC substrates. As a result, a silicon layer with Si–O–Si bonds, as well as monolayers with C–Si–O bonds, are formed on RC surfaces, resulting in hydrophobic and interconnected silicon coating on the RC substrates [46]. In comparison with the pristine RC film, the high-resolution XPS spectra O 1s and C 1s of the MTS/RC-60 showed two new peaks at 532.2 eV (Si–O–Si) and 284.4 eV (C–Si–O) (Figure 4C,D), clearly indicating the successful surface silanization on the MTS/RC-60 film [42,58,59,60]. After being modified by MTS, the bond energy of C–O–C/C–O–H shifted to 531.8 eV (Figure 4C) [61,62]. Additionally, the bond energy of C–C/C–H and C–O–H/C–O–C shifted to 285.0 and 286.6 eV, respectively (Figure 4D) [60,63,64,65,66]. XPS can be utilized to analyze the elemental composition of the surface in the range of 1–10 nm. It is important to note that the peaks of O and C present in pristine RC films were still observed on the MTS/RC-60 film (Figure 4C,D), indicating that a thin polysiloxane layer with a thickness below 10 nm formed on the RC surface.

**Figure 5:**
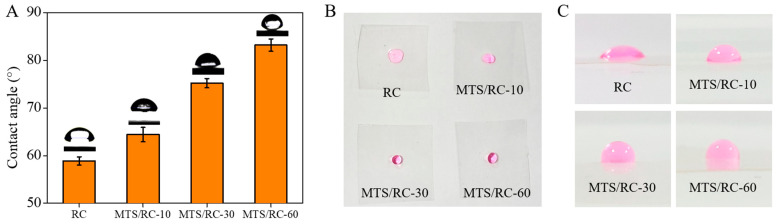
(**A**) Water contact angle values for the pristine RC film and the MTS/RC-10, MTS/RC-30, and MTS/RC-60 films. (**B**,**C**) Photographs of (**B**) the top and (**C**) the side of a 10 µL rhodamine-B-dyed water drop (10 mg/mL) that was dropped on the surface of the pristine RC film and the RC/MTS films.

We next examined the effects of the MTS deposition time on the wettability of the obtained MTS/RC films by measuring the water contact angle. The water contact angle was 58.9 ± 0.86° for the pristine RC film. After MTS modification, the water contact angles were found to be 64.4 ± 1.5, 75.2 ± 1.0, and 83.2 ± 1.3° for the RC/MTS-10, RC/MTS-30, and RC/MTS-60 films, respectively (Figure 5A). These results suggest that the presence of methyl groups in MTS endows RC films with hydrophobicity [47,67,68,69,70,71]. To clearly visualize the difference in wettability between various films, we dropped a colored aqueous solution on the pristine RC film and the MTS/RC films (Figure 5B). As expected, the MTS/RC-30 and MTS/RC-60 films exhibited a higher hydrophobicity than the pristine RC film and the MTS/RC-10 film.

**Figure 6:**
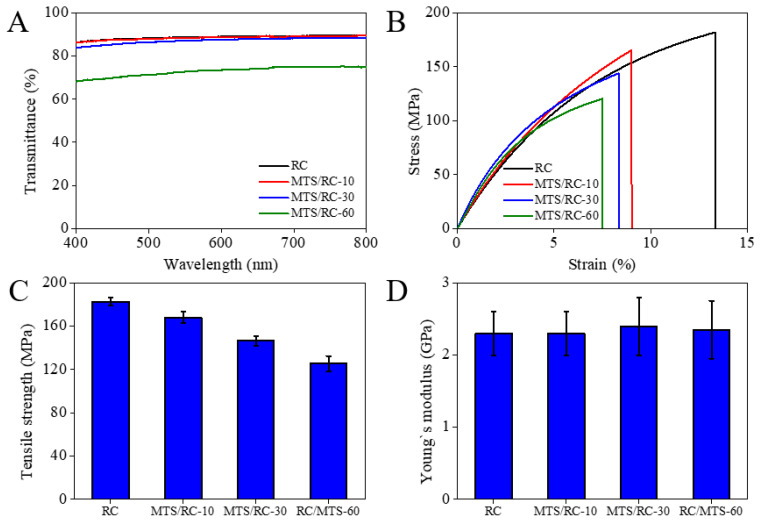
(**A**) Optical transmittance of the pristine RC film and the MTS/RC-10, MTS/RC-30, and MTS/RC-60 films. (**B**–**D**) Mechanical properties of the pristine RC film and the MTS/RC films. (**B**) Stress–strain curve, (**C**) tensile strength, and (**D**) Young’s modulus of the pristine RC film and the MTS/RC-10, MTS/RC-30, and MTS/RC-60 films.

As can be observed in Figure 6A, the transmittance of the MTS/RC-10 and MTS/RC-30 films remained almost completely unchanged compared with that of the pristine RC film. The transmittance of the MTS/RC films at 550 nm decreased from 88.7% to 87.0% as the MTS deposition time was increased to 30 min. Though the transmittance of the MTS/RC-60 film at 550 nm was determined to be 72.8%, this film still preserved optical transparency. It is important to note that the optical transmittance of MTS/RC films was comparable to that of previously reported biopolymer (chitosan, PLA, etc.)-based films [72,73,74,75]. Recently, Makarov et al. reported N-methylmorpholine-N-oxide (NMMO) surface treatment based on the partial dissolution of cellulose and its coagulation for modification of a paper surface [30]. In general, surface-modified RC films exhibit uniform surface morphology as well as high transmittance compared with surface-modified papers, probably due to a perfect dissolution and regeneration process. As shown in Figure 6B–D, the mechanical properties of the pristine RC film and the MTS/RC films were investigated by conducting tensile stress–strain measurements as a function of the MTS deposition time. The pristine RC film exhibited the highest tensile strength (182.6 MPa) among all the tested films. The tensile strength of the MTS/RC-30 and -60 films was found to be considerably lower, namely 146.4 and 125.9 MPa, respectively [76,77]. The decrease in tensile strength may have been due to the hydrochloric acid produced as a by-product of silanization. The acidic degradation of the cellulose chains can cause a decrease in MTS/RC film strength [51,78,79]. However, the pristine RC film and the MTS/RC films exhibited a nearly identical Young’s modulus (2.3 ± 0.4 GPa). It is important to note that the mechanical strength of the MTS/RC films is superior to those of previously reported nanocellulose-based hydrophobic films (48.8‒103.7 MPa), PLA-based hydrophobic films (~63.2 MPa), and hydrophobic paper composites (16‒20 MPa) [51,53,80,81].

**Figure 7:**
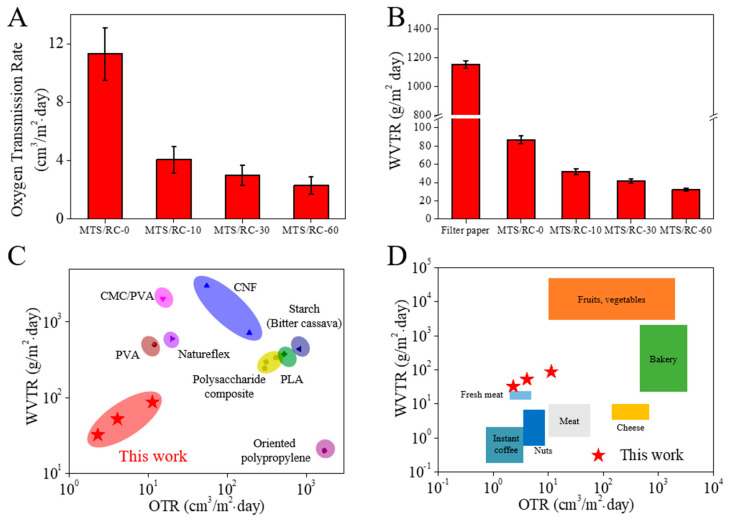
(**A**) OTR values and (**B**) WVTR values for the filter paper, pristine RC film, and MTS/RC films for various MTS reaction times. (**C**) Comparison of the OTR and WVTR of the reported food packaging films and MTS/RC films (red stars). (**D**) OTR and WVTR requirements for different food products and the OTR and WVTR of the MTS/RC films (red stars).

To examine the effect of the MTS coating on the barrier properties, we measured the OTR and WVTR of the filter paper, the pristine RC film, and the MTS/RC films (Figure 7A,B). The pristine RC film exhibited an OTR of 11.3 ± 1.8 cm^3^/m^2^ per day, while the MTS/RC-10, MTS/RC-30, and MTS/RC-60 films exhibited an OTR of 4.1 ± 0.9, 3 ± 0.7, and 2.3 ± 0.6 cm^3^/m^2^ per day, respectively. The WVTR values of the filter paper and the pristine RC film were 1152.8 and 86.8 g/m^2^ per day at 23 °C and 50% RH, respectively. On the other hand, the MTS/RC-10, MTS/RC-30, and MTS/RC-60 films exhibited WVTR values of 52.1 ± 3.1, 41.7 ± 2.1, and 32.1 ± 1.3 g/m^2^ per day, respectively. These findings suggest that the MTS coating on the RC films could reduce the OTR and WVTR of the pristine RC film by 80% and 63%, respectively. The low OTR and WVTR of the MTS/RC films could be attributed to the hydrophobic MTS layer as well as its compact structure on the surface of the RC film [82,83]. These data are in line with the contact angle results presented in Figure 5. It is worth noting that the MTS/RC films have considerably superior barrier properties compared with those of previously reported natural polymer (CNF, starch, PLA, Natureflex, polysaccharide composite, etc.)-based films (Figure 7C) [84,85,86,87,88,89]. The oxygen and moisture barrier performance of the obtained MTS/RC films meets the packaging requirements for bakery products, fruits, and vegetables (Figure 7D).

## 4. Conclusions

In summary, we utilized a one-step CVD method to fabricate a high-performance hydrophobic bioplastic film that is optically transparent, mechanically strong, and has a good barrier performance. The interconnected silicon coating of both a silicon layer and a monolayer deposited on an RC surface via a gas–solid reaction led to the enhancement in hydrophobicity and barrier properties. The obtained MTS/RC films exhibited good mechanical properties as well as good oxygen/water vapor barrier performance. Notably, the hydrophobic MTS/RC-30 film possessed an optical transparency of 87% at 550 nm, a high tensile strength of 146 MPa, a low OTR of 3 cm^3^/m^2^ per day and a low WVTR of 41 g/m^2^ per day, which are superior to those of other hydrophobic biopolymer films reported in previous studies. These findings may be valuable for the production of high-performance natural polymer-based films.

## Data Availability

Data are contained within the article.
